# Fucoidan alleviated colitis aggravated by fiber deficiency through protecting the gut barrier, suppressing the MAPK/NF-κB pathway, and modulating gut microbiota and metabolites

**DOI:** 10.3389/fnut.2024.1462584

**Published:** 2025-01-24

**Authors:** Weiyun Zheng, Shuangru Tang, Xiaomeng Ren, Shuang Song, Chunqing Ai

**Affiliations:** ^1^School of Agronomy and Life Science, Shanxi Datong University, Datong, China; ^2^National Engineering Research Center of Seafood, School of Food Science and Technology, Dalian Polytechnic University, Dalian, China; ^3^National & Local Joint Engineering Laboratory for Marine Bioactive Polysaccharide Development and Application, Dalian Polytechnic University, Dalian, China

**Keywords:** polysaccharide, intestinal diseases, gut microbiota, metabolites, IBD susceptibility

## Abstract

Insufficient dietary fiber intake has become a global public health issue, affecting the development and management of various diseases, including intestinal diseases and obesity. This study showed that dietary fiber deficiency enhanced the susceptibility of mice to colitis, which could be attributed to the disruption of the gut barrier integrity, activation of the NF-κB pathway, and oxidative stress. *Undaria pinnatifida* fucoidan (UPF) alleviated colitis symptoms in mice that fed with a fiber deficient diet (FD), characterized by increased weight gain and reduced disease activity index, liver and spleen indexes, and histological score. The protective effect of UPF against FD-exacerbated colitis can be attributed to the alleviation of oxidative stress, the preservation of the gut barrier integrity, and inhibition of the MAPK/NF-κB pathway. UPF ameliorated the gut microbiota composition, leading to increased microbiota richness, as well as increased levels of *Muribaculaceae*, *Lactobacillaceae*, and *Bifidobacterium* and reduced levels of *Proteobacteria*, *Bacteroidetes*, and *Bacteroides*. Metabolomics analysis revealed that UPF improved the profile of microbiota metabolites, with increased levels of carnitine and taurine and decreased levels of tyrosine and deoxycholic acid. This study suggests that UPF has the potential to be developed as a novel prebiotic agent to enhance human health.

## 1 Introduction

Inflammatory bowel disease (IBD) is a chronic condition manifesting as recurring inflammation in the intestine. IBD encompasses two primary subtypes: Crohn’s disease and ulcerative colitis (UC), and it is emerging as a significant global public health issue (1). As a refractory form of IBD, UC is characterized by high incidence, prolonged duration, and frequent recurrences, with classic symptoms including abdominal pain, diarrhea, and hematochezia (2). The pathogenesis of UC is very complex and multifactorial, with diet playing a vital role as a significant environmental factor in determining the progression of UC (3). Epidemiological data has identified low dietary fiber intake as a significant risk factor for UC, and its precise mechanism can be attributed to its impact on the mucosal barrier, gut microbiota, and immune response (4).

The primary clinical treatment options for UC include medications, e.g., anti-inflammatory agents and immunomodulators, as well as surgical removal. However, the inevitable side effects of first-line therapies present a great challenge in the treatment of UC, making it necessary to explore innovative strategies for treating UC or their use as an important adjunct to drug treatment. Accumulating studies have shown that some bioactive components in food exhibit beneficial effects on symptoms of UC, e.g., polysaccharides, prebiotics, and probiotics (5, 6). Fucoidan can inhibit colonic inflammation induced by dextran sulfate sodium (DSS) and promote intestinal barrier repair. Microbes-mediated modulation of bile acid metabolism and butyrate production are identified as crucial mechanisms underlying the therapeutic intervention of fucoidan in alleviating colitis (7). Oat β-glucan supplementation effectively alleviated colitis symptoms in mice by modulating the gut microbiota and metabolites and protecting the integrity of epithelial tight junctions (8).

The gastrointestinal tract, harboring trillions of microorganisms, functions as an imperceptible metabolic “organ” that exerts a crucial influence on the health of the host. The findings from studies on germ-free mice have demonstrated the indispensable role of the intestinal flora in the maturation and progression of mucosal and systemic immune systems (9). In comparison to conventional animal models, germ-free animal models elucidate the pivotal roles of gut microbes in shaping the structure and functionality of gastrointestinal tract (10). Gut microbes contain more genes than human genomes, which can participate in various metabolic processes, such as nutrient acquisition (11). Therefore, the gut microbiota and the host maintain a symbiotic relationship, and any alterations can contribute to the development of various diseases in the host (12). Hence, it suggests that the regulation of the interaction between the gut microbiota and the host by polysaccharides may be a potential action mechanism for ameliorating IBD.

*Undaria pinnatifida* is rich in some bioactive substances, and fucoidan (UPF) is one of the most important ingredients. UPF effectively decreased weight gain in mice that fed with a high-fat diet by modulating the gut microbiota composition and ameliorating lipid abnormalities induced by excessive fat (13). Additionally, UPF exhibits other beneficial effects on the body, e.g., anti-diabetic, antitumor, and anti-inflammatory activities. However, the beneficial effect of UPF on UC and its mechanism remain unclear, particularly in the context of dietary fiber deficiency. This study demonstrated that FD exacerbated colitis, while UPF alleviated colitis by suppressing the MAPK/NF-κB pathway, protecting the gut barrier integrity, and modulating the gut microbiota and metabolites. It suggests that UPF has the potential to be developed as a novel prebiotic dietary fiber for augmenting human health.

## 2 Materials and methods

### 2.1 Materials

DSS (purity of 98%, molecule weight: 36 kDa–50 kDa) was provided by Yeasen Biotechnology Co., Ltd. (Shanghai, China). β-actin (4,970), p-p65 (3,033), ZO-1 (8,193), claudin-1 (4,933), p65 (8,242), p-p38 (8,690), p38 (4,511), JNKs (4,511), p-JNKs (8,690), p-ERKs (8,690), ERKs (4,511), p-IκBα (2,859), and TLR4 (38,519) were provided by Cell Signaling Technology (MA, United States), and MyD88 (ab133739), occludin (ab216327), IκBα (ab76429), and goat anti-rabbit IgG (ab6721) were obtained from Abcam (MA, United States). FD was provided by HFK Bioscience Co., Ltd. (Supplementary Table S1, Beijing, China) (14). Tumor necrosis factor-α (TNF-α), lipopolysaccharide (LPS), interleukin-1β (IL-1β), and IL-10 were obtained from Enzyme-linked Biotechnology Co., Ltd. (Shanghai, China).

### 2.2 Preparation of UPF

*U. pinnatifida* was purchased from a local market in Dalian, Liaoning, China. The preparation of UPF was performed based on our previous method (13), and its structure information, including molecular weight, monosaccharides composition, and the levels of uronic acid, sulfate group, and protein, was presented in Supplementary Figure S1.

### 2.3 Mice experiments

C57BL/6 mice (18–20 g, SPF, male, and 7 weeks old) were obtained from Liaoning Changsheng Biotechnology Co., Ltd. (Benxi, Liaoning, China). All animal care and protocols were carried out in strict adherence to the guidelines of the National Institutes of Health for the use and care of laboratory animals, and this study received the approval from the Animal Ethics Committee of Dalian Polytechnic University (DLPU2022016). Mice were housed at the Laboratory Animal Center of Dalian Polytechnic University and had unrestricted access to food and water. At the end of the mice experiments, blood was collected from the ocular region after administering 1.4% isoflurane for inhalation anesthesia. Isoflurane was carried by pure O_2_ at a steady flow rate of 1.0 L/min. Mice were euthanized by cervical dislocation for organ isolation.

#### 2.3.1 Experiment 1

**Figure 1:**
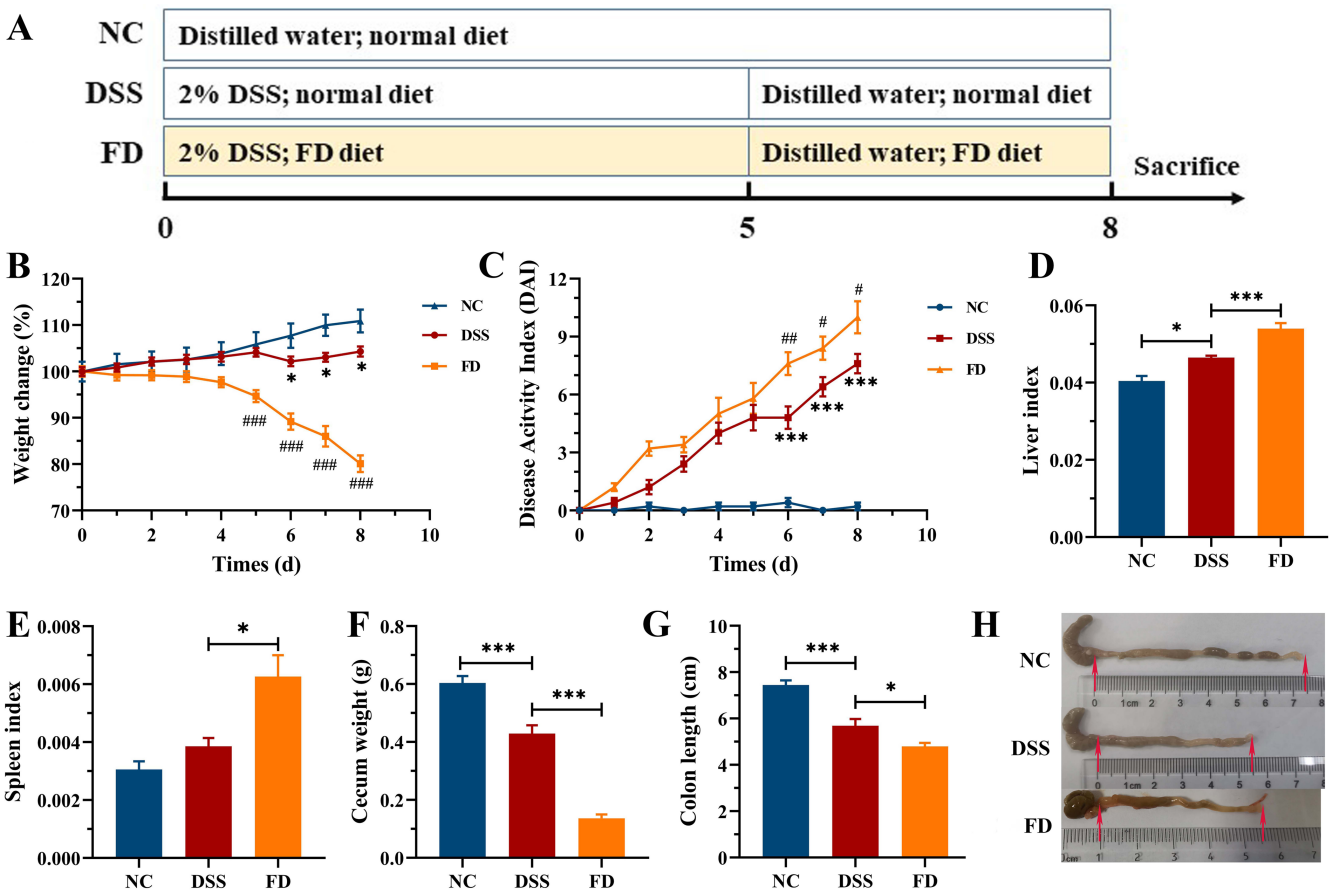
Effect of FD on symptoms of colitis induced by DSS (*n* = 7/group). Procedure of mice experiment **(A)**. Body weight **(B)**. DAI **(C)**. ^*^p < 0.05 and ^***^p < 0.001 vs. the NC group; ^#^*p* < 0.05, ^##^*p* < 0.01, and ^###^*p* < 0.001 vs. the DSS group. Liver index **(D)**. Spleen index **(E)**. Cecum weight **(F)**. Colon length **(G)**. Macroscopic examination of the colon **(H)**.

Mice were divided into three groups (*n* = 7/group, Figure 1A) at a randomized manner: (1) normal control group (NC): normal feeding for 8 days; (2) DSS group: 2% (w/v) of DSS for 5 days, normal water for 3 days, and normal diet for 8 days; (3) FD group: 2% of DSS for 5 days, normal water for 3 days, and FD for 8 days. The liver, spleen, and colon were measured after mice sacrificed.

#### 2.3.2 Experiment 2

**Figure 2:**
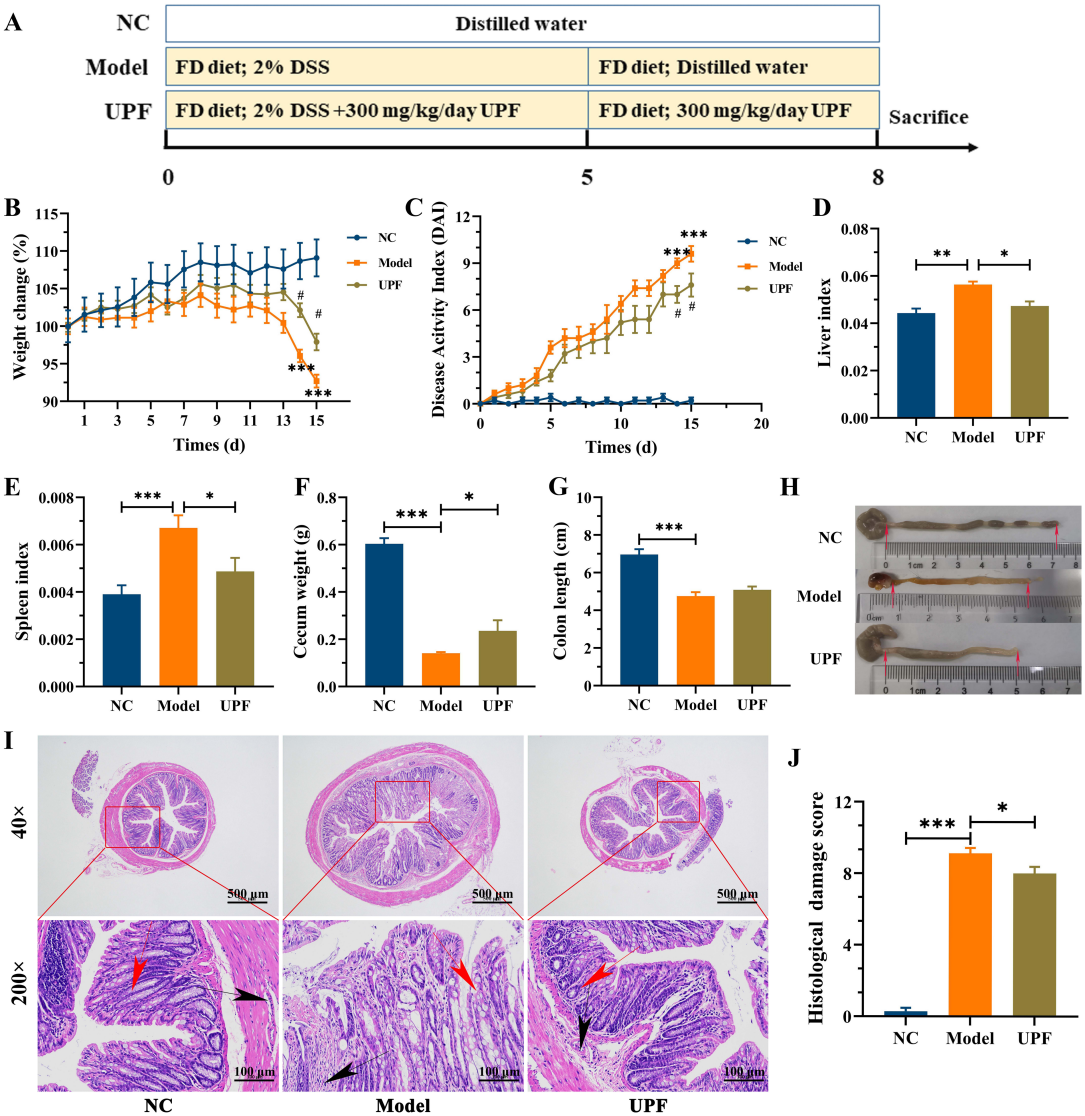
Effect of UPF on symptoms of FD-aggravated colitis (*n* = 7/group). Schematic diagram of mice experiment **(A)**. Body weight **(B)**. DAI **(C)**. ^***^p < 0.001 vs. the NC group; ^#^*p* < 0.05 vs. the model group. Liver index **(D)**. Spleen index **(E)**. Cecum weight **(F)**. Colon length **(G)**. Macroscopic examination of the colon **(H)**. Histological analysis based on HE staining **(I)**, scale bar = 500 μm (up), scale bar = 500 μm (down). The red arrows indicate the presence of crypts, the black arrows indicate the infiltration of inflammatory cells. Histological score, *n* = 3/group **(J)**.

As illustrated in Figure 2A, mice were divided into three groups (*n* = 9/group): normal group (NC), DSS + FD group (model), and DSS + FD + UPF group (UPF). The NC group were provided with normal diet and water, and the other two groups were given 2% of DSS for 5 days, water for 3 days, and FD for 8 days. Meanwhile, the UPF group were orally administrated with 300 mg/kg/day of UPF for 8 days. Body weight and disease activity index (DAI) were monitored using the assessment system established in previous study (Supplementary Table S2) (15). At the end of mice experiment, the liver, spleen, cecum, and colon were measured, and the liver and spleen indexes were calculated using a previously established method (7).

### 2.4 Histological analysis

According to standard procedure, colon tissues were fixed, dehydrated, embedded, sectioned, and stained with hematoxylin and eosin (HE), Alcian blue (AB), and periodic acid–Schiff (PAS). The images were visualized using a light microscope (Nikon, Japan) and then analyzed following the previously described method (15).

### 2.5 Biochemical assays

The colon tissues were weighed and homogenized in nine times of PBS solution (w/v), and then the supernatants were collected after centrifugation. The contents of malondialdehyde (MDA), inducible nitric oxide synthase (iNOS), total superoxide dismutase (T-SOD), myeloperoxidase (MPO), and catalase (CAT) in the supernatants were measured using kits from Nanjing Jiancheng Bioengineering Institute (Nanjing, China). The levels of TNF-α, IL-1β, IL-10, and LPS were determined using ELISA kits according to manufacturer’s instructions.

### 2.6 Western blotting

The excised colon samples were thoroughly homogenized in RIPA lysis buffer to facilitate protein extraction, followed by quantification using the BCA kit (Solarbio, China). Protein samples were separated using SDS-PAGE and transferred onto the methanol-activated prefabricated polyacrylamide (PVDF) membrane. After being blocked with 5% skim milk for 1 h, the membranes were incubated overnight at 4°C with the diluted primary antibodies, followed by incubation with the secondary antibodies (listed in the Supplementary Table S4) at room temperature for 1 h. Subsequently, they were treated with the chemiluminescence detection substrate (Beyotime, China). The densities of target bands were visualized using a ChemiDoc Touch Imaging System (Bio-Rad, United States) and analyzed using ImageJ software (Java 1.8.0, NIH; Bethesda, MD, United States).

### 2.7 Targeted analysis of microbiota metabolites using HPLC-QTRAP/MS

The preparation of fecal samples was conducted as described in previous study (16). The analysis of microbiota metabolites was carried out in an ABSCIEX 4000 QTRAP (United States) that coupled to a HPLC (Shimadzu, Japan). The methodologies that used for data collection, processing, and analysis were consistent with those in previous study (16).

### 2.8 Sequencing analysis of fecal microbiota community

DNA extraction and sequencing analysis were performed at Novogen Biological Technology Co., Ltd. (Beijing, China) (13). Shannon and Chao1 indexes were employed to assess microbiota diversity and richness. Principal coordinate analysis (PCoA) and unweighted pair-group method with arithmetic means (UPGMA) were employed to assess the similarities in the microbiota communities between the groups. The bacterial taxa that exhibited significant differences among the groups was identified using linear discriminant analysis (LDA).

### 2.9 Immunohistochemical analysis

After rinsing the dewaxed sections with distilled water, they were subjected to citric acid antigen retrieval solution for repair, followed by sequential incubation in 3% H_2_O_2_ and 3% BSA solution. Then, the sections were incubated with the diluted primary antibodies and secondary antibody (listed in the Supplementary Table S4). After washing with PBS (pH 7.4), the slices were stained by DAB and hematoxylin. The expression of proteins was examined using a light microscope (Nikon, Japan). The images were analyzed using ImageJ software (Java 1.8.0, NIH; Bethesda, MD, United States).

### 2.10 Statistical analysis

Data are presented as means ± SEM. Significant difference among groups was determined using one-way analysis of variance, followed by post-hoc Tukey multiple comparisons test. GraphPad Prism 8.0 (La Jolla, United States) was used for data analysis. Spearman analysis was used to establish the correlations between key metabolites and biochemical indexes.

## 3 Results and discussion

### 3.1 FD exacerbated the symptoms of colitis in DSS-treated mice

Histopathological alterations that found in DSS-induced colitis closely resemble those in human UC, rendering that this model is a valuable tool for investigating the pathogenesis of colitis (17). The pathogenesis of UC is multifactorial, and low dietary fiber intake is believed to have an obvious impact on its onset and progression (18). The impact of FD on colitis was initially assessed in mice treated with 2% DSS (Figure 1). Mice in the model group exhibited obvious colitis symptoms, characterized by a reduction in weight gain, cecum weight, and colon length, as well as an increase in the liver index and DAI. FD exacerbated aforementioned symptoms of colitis, and there was an obvious increase in the spleen index. It suggests that FD may increase the susceptibility of human to IBD. It is similar to the highly susceptibility of mice fed with a no-fiber diet to colitis induced by DSS (19). Considering that dietary fiber intake is far below the recommended level by WHO (20), a colitis mouse model with FD can be more suitable for investigating the therapeutic strategies of colitis.

### 3.2 FD exacerbated intestinal inflammation by activating the NF-κB pathway

**Figure 3:**
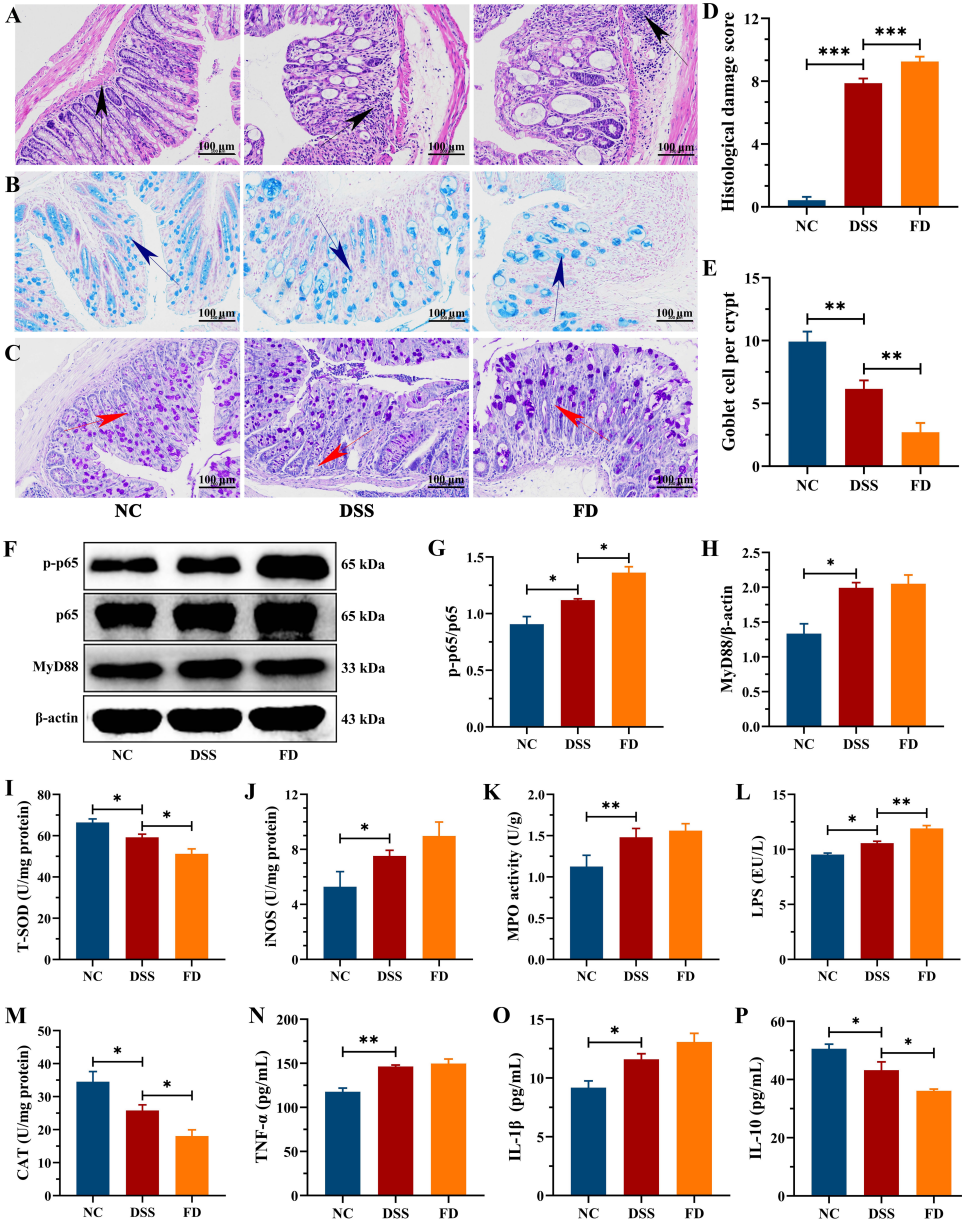
FD aggravated colonic inflammation in mice. Representative images of the colon stained with HE, AB, and PAS **(A–C)**. Scale bar = 100 μm. Black arrows indicate inflammatory infiltration, blue arrows denote colonic mucus, and red arrows indicate crypt damage. Histological score, *n* = 3/group **(D)**. The amount of goblet cells per crypt, *n* = 3/group **(E)**. Representative images of p-p65, p65, and MyD88 in the colon tissues and their relative content, *n* = 3/group **(F–H)**. The contents of LPS, CAT, T-SOD, iNOS, and MPO in the colon, *n* = 5/group **(I–M)**. TNF-α **(N)**, IL-1β **(O)**, and IL-10 **(P)**, *n* = 5/group.

HE staining revealed significant colon damage in the DSS group, characterized by inflammatory cell infiltration, loss of crypts, and erosion of mucosa, resulting in a high histological score (Figures 3A,D). AB and PAS staining revealed disruptions in the integrity of the mucus layer in the DSS group, characterized by a reduction in the number of goblet cells, along with a notable decrease in glycoproteins density, and thinner mucous thickness (Figures 3B,C,E). The levels of TNF-α and IL-1β were higher in the DSS group compared to the NC group, and IL-10 was lower (Figures 3N–P). It indicated that DSS treatment resulted in colonic inflammation, which is consistent with previous study (17). FD exacerbated colitis, as evidenced by increased crypts loss, inflammatory infiltration, and IL-1β level, while also resulting a decrease in IL-10 level and mucus layer thickness. Recent study showed that FD increased the relative abundance of mucus-eroding bacteria that degrade the mucus layer, leading to greater epithelial access (14). Moreover, FD increased the expression level of chemokine *Cxcl2* and promoted the interaction between neutrophils and endothelial cells, promoting an inflammatory state (19). These results suggest an association between FD and the initiation and progression of IBD.

NF-κB consists of five distinct proteins, including p65 and p50, and it is one of the key regulators in the immune system. In individuals diagnosed with IBD, the activation of this pathway orchestrates the cytokines profile that foster inflammation (21). MyD88, as a central hub, plays a crucial role in the inflammatory pathway, functioning as an adaptor in the downstream inflammatory signaling pathway of Toll-like receptor and IL-1 receptor. Its primary function is to facilitate the activation of the NF-κB pathway (22). The NF-κB pathway can be triggered by diverse stimuli, e.g., LPS (23). DSS distinctly upregulated the expression levels of p-p65/p65, MyD88, and LPS compared to the NC group (Figures 3F–H,L). FD increased the levels of p-p65/p65 and LPS, implying that FD can exacerbate colonic inflammation by activating the NF-κB pathway. This could be attributed to FD’s disruption of micro-ecological equilibrium and its impact on intestinal permeability (24).

### 3.3 FD aggravated oxidative stress in the colon tissues of mice with colitis

Serious mucosal injury can induce the generation and release of reactive oxygen species (ROS), in addition to increasing inflammatory cell infiltration. Overproduction of ROS disrupts the delicate balance between oxidants and antioxidants, which is crucial for the recurrence and progression for IBD (25). The contents of iNOS and MPO were significantly higher in the DSS group than the NC group, whereas CAT and T-SOD activities were reduced (Figures 3I–K,M). CAT and T-SOD are pivotal constituents of the antioxidant defense network, playing crucial roles in the clearance of ROS (26). MPO restricts the utilization of nitric oxide (NO) by generating reactive oxidants, e.g., hypochlorous acid, which can contribute to endothelial dysfunction (27). NO is a key biological mediator, and its overproduction that catalyzed by iNOS can be cytotoxic (28). FD decreased CAT and T-SOD levels and increased iNOS level, thereby aggravating colonic inflammation.

### 3.4 UPF ameliorated the symptoms of colitis exacerbated by FD

As mentioned above, significant colitis symptoms were observed in the model group, including a reduction in weight gain, colon length, and cecum weight, and an increase in spleen index, liver index, DAI, inflammatory infiltration, and tissue damage (Figure 2). UPF increased weight gain and cecum weight and reduced DAI, liver index, and spleen index in mice. UPF alleviated inflammatory response, as evidenced by a reduction in inflammatory infiltration, crypts loss, colonic edema, and histological score. This is similar with that fucoidan increased colon length and reduced spleen index, DAI, and colonic inflammation in mice (7). Fucoidan from *Macrocystis pyrifera* reduced DAI, weight loss, and spleen index and increased colon length in colitis mice (29). It implies that UPF can serve as a prebiotic dietary fiber for the maintenance of gut health.

### 3.5 UPF inhibited the MAPK/NF-κB pathway and oxidative stress

**Figure 4:**
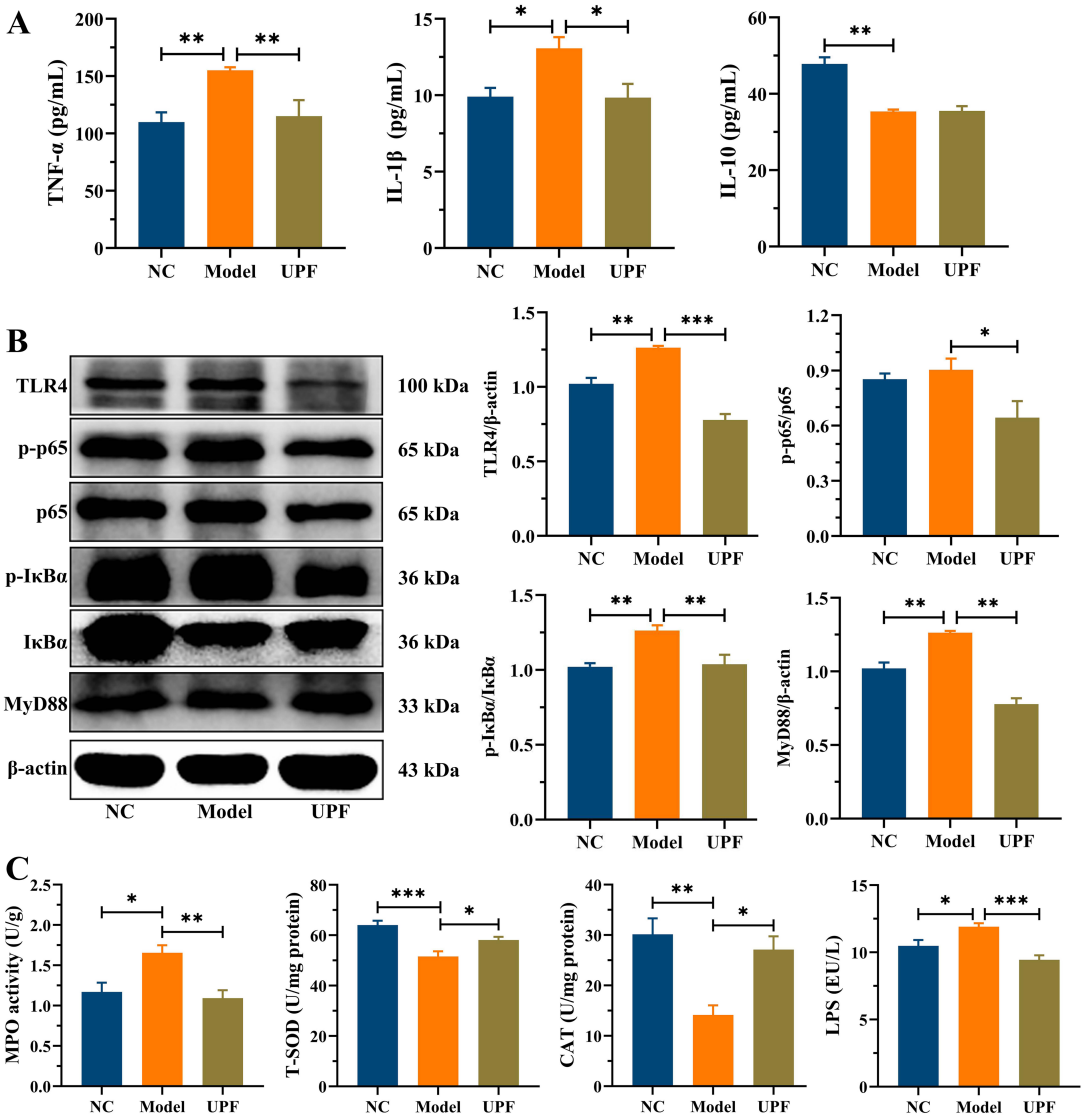
Inhibition effect of UPF on colonic inflammation. The contents of TNF-α, IL-1β, and IL-10, *n* = 5/group **(A)**. Representative images of TLR4, p-p65, p65, p-IκBα, IκBα, and MyD88 in the colon and their relative levels, *n* = 3/group **(B)**. The levels of MPO, T-SOD, CAT, and LPS in the colon, *n* = 5/group **(C)**.

**Figure 5:**
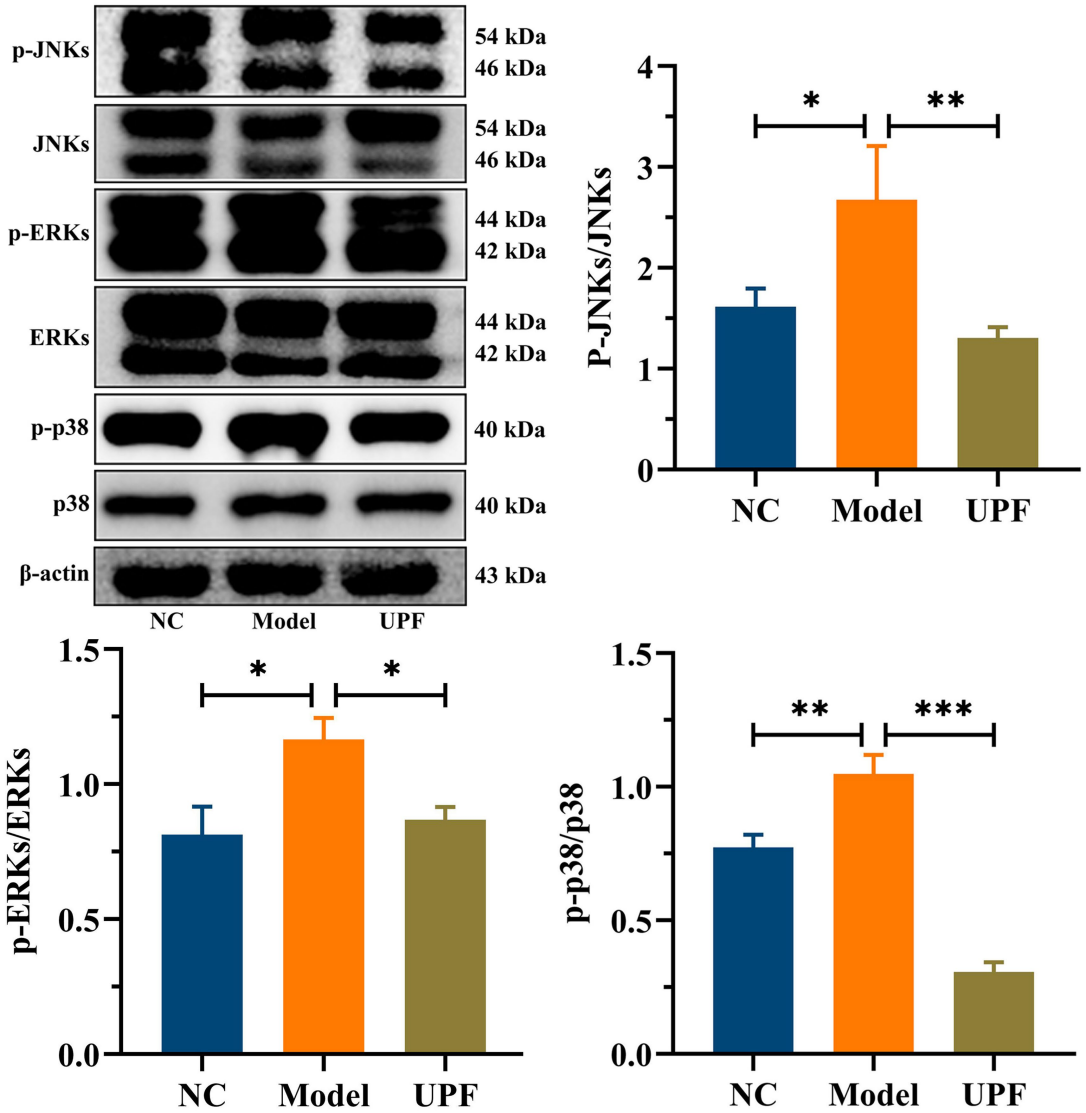
Inhibition effect of UPF on MAPK pathway. Representative images of p-JNKs, JNKs, p-p38, p38, p-ERKs, and ERKs in the colon and their relative levels, *n* = 3/group.

The levels of pro-inflammatory cytokines TNF-α and IL-1βwere significantly higher in the model group than those in the NC group, while there was a notable reduction in IL-10 (Figure 4A). UPF had no effect on IL-10 but reduced the levels of IL-1β and TNF-α, indicating its anti-inflammatory activity. To explore its potential mechanism, we evaluated the effect of UPF on the pivotal proteins of the MAPK/NF-κB pathway and oxidative stress-associated markers. UPF significantly decreased the levels of p-IκBα/IκBα, TLR4, and MyD88 in DSS-treated mice (Figure 4B). It implied that UPF can inhibit the activation of the NF-κB pathway to alleviate colitis. This is consistent with that polysaccharides from *Scutellaria baicalensis* alleviated colonic inflammation via the inhibition of the NF-κB pathway (30). UPF ameliorated the imbalance between oxidative stress and antioxidant defense by increasing CAT level, which break down H_2_O_2_ into water and oxygen, enhancing T-SOD activity to neutralize ROS, and reducing MPO level (Figure 4C). This suggests that UPF can reduce colonic inflammation by alleviating oxidative stress. In addition, UPF reduced LPS level in the gut, thereby mitigating colonic inflammation by attenuating the infiltration of inflammatory factors (23). This is consistent with that grape peel powder attenuated TNBS-induced inflammatory responses and oxidation via modulation of the NF-κB pathway and antioxidant oxidase levels (31). MAPKs, including JNKs, ERKs, and p38, are intracellular signal transduction factors that exert key regulatory roles in pro-inflammatory mediators synthesis, as well as cellular processes encompassing proliferation, differentiation, and apoptosis (32). The MAPK pathway can be activated during colitis development, leading to an augmented production of pro-inflammatory cytokines (33). The ratios of p-JNKs/JNKs, p-p38/p38, and p-ERKs/ERKs were distinctly increased in the model group, indicating that the MAPK pathway was activated (Figure 5). UPF treatment reduced the levels of p-p38/p38, p-JNKs/JNKs, and p-ERKs/ERKs, implying that UPF can suppress inflammatory response by blocking the transmission of this pathway. It is consistent with *Mesona chinensis* polysaccharides alleviated colonic inflammation by suppressing the activation of the MAPK/NF-κB pathway (34).

### 3.6 UPF protected against the disruption of the gut barrier integrity

**Figure 6:**
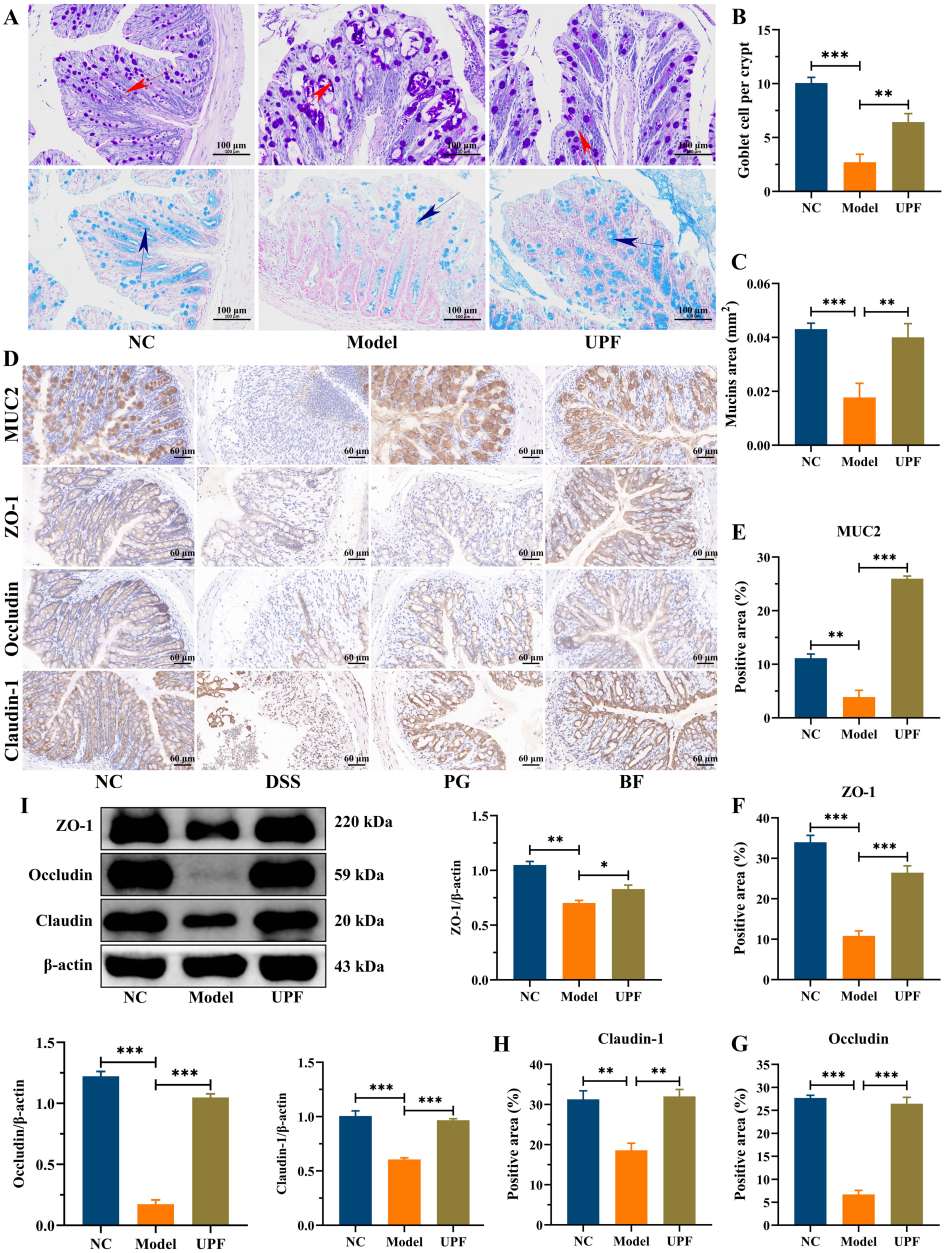
Protective effect of UPF on the gut barrier integrity. AB/PAS staining **(A)**, number of goblet cells per crypt **(B)**, and mucin area **(C)**, scale bar = 100 μm, *n* = 3/group. Immunohistochemistry staining and analysis, including MUC2, ZO-1, occludin, and claudin-1 **(D–H)**, scale bar = 60 μm, *n* = 3/group. Representative images of ZO-1, occludin, and claudin-1 in the colon and their relative levels, *n* = 3/group **(I)**.

The intestinal tract harbors the largest barrier between hostile environment and the internal milieu, and its defects are related to various diseases, including IBD and other extra-intestinal disorders (35). The intestinal barrier is a robust defense system against detrimental substances, effectively segregating the gut, which is filled with microbes and nutrients, from the underlying sterile tissue. It primarily comprises the mucus layer, the epithelial layer, and the underlying lamina propria (36). The mucus layer integrity was disrupted in the model group, characterized by a reduction in goblet cells, crypts loss, and thinning of the mucus layer (Figures 6A–E). In contrast, UPF mitigated crypts loss and the reduction in goblet cells and glycoproteins, thereby safeguarding the mucus layer integrity. Tight junction proteins (TJs) establish intercellular connections between adjacent epithelial cells and regulate the paracellular selective permeability to solutes (36). TJs are a protein complex, including some claudin members and occludin, along with cytosolic proteins (ZO-1) that link to the actin cytoskeleton. In this study, the distribution and expression of TJs and MUC2 in mice colon were investigated by IHC. The brown and light brown sections were regarded as the positive expression. In the model group, claudin-1, occludin, and ZO-1 in mice colon were distinctly depleted when compare with those in NC group (Figures 6D–H). The disruption of intestinal epithelial junctions can result in enhanced permeability of the epithelium, facilitating the translocation of toxic substances, e.g., LPS. UPF upregulated the expression levels of ZO-1, claudin-1, and occludin to maintain the integrity of intestinal epithelial tight junctions, resulting in a reduction in the invasion of toxic substances (Figure 6I). It is consistent with that β-glucan mitigated colonic inflammation in mice associated with its effect on the mucus layer and intestinal epithelial junctions (8).

### 3.7 UPF modulated alterations in the gut microbiota in mice with colitis

**Figure 7:**
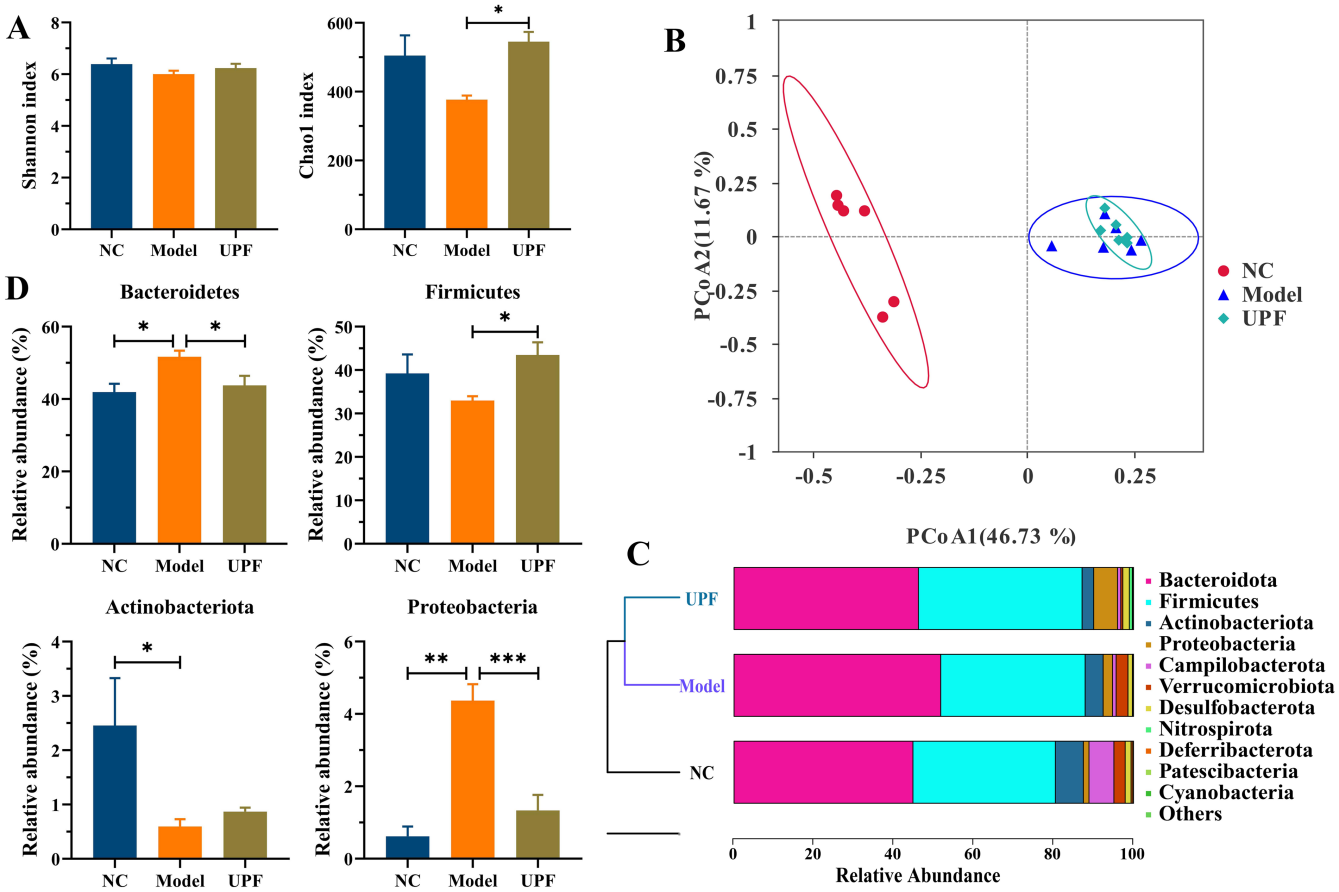
Modulation effect of UPF on the gut microbiota (*n* = 5/group). Chao1 and Shannon indexes **(A)**. PCoA **(B)**. UPGMA **(C)**. Relative abundances of *Bacteroidetes*, *Firmicutes*, *Proteobacteria*, and *Actinobacteria* at the phylum level **(D)**.

In the pathogenesis of IBD, there is a bidirectional relation between alterations in disease status and changes in the composition and functionality of the gut microbiota, while dysregulation of the gut microbiota is characterized by diminished biodiversity and changes in microbial community and spatial distribution (37). The model and UPF groups had no difference on the Shannon index (Figure 7A). The Chao1 index was decreased in the model group (*p* > 0.05), while it was increased in the UPF group, implying that UPF can enhance microflora richness. PCoA and UPGMA revealed distinct separation between the NC and model groups, while no difference was found between the UPF and model groups (Figures 7B,C). Feruloylated oligosaccharides alleviated colitis by increasing microbiota richness and species diversity of beneficial bacteria, e.g., *Roseburia* and *Akkermansia* (38). The efficacy of fecal microbiota transplantation in UC patients was positively associated with microbial diversity (39). It suggests that UPF could benefit the gut health or prevent the development of colitis by increasing microbiota richness.

The levels of *Bacteroidetes* and *Proteobacteria* were significantly higher in the model group than the NC group, while *Firmicutes* (*p* > 0.05) and *Actinobacteria* were lower (Figure 7D). In contrast, the levels of *Proteobacteria* and *Bacteroidetes* were reduced in the UPF group, while *Firmicutes* level was increased. *Firmicutes* and *Bacteroidetes* are the two predominant bacterial phyla, constituting over 90% of overall gut microbiota in the human gut, and their ratio has been linked to a range of pathologies, including IBD and obesity (40). The fluctuation of *Firmicutes* level and microbiota diversity in IBD is closely associated with disease status (41). Some probiotics can manage IBD by increasing *Firmicutes* and reducing *Bacteroidetes* (40). *Proteobacteria* as an indicator of microbial dysregulation represents a potential diagnostic criterion for diseases. An increase of *Proteobacteria* members has been linked to IBD, e.g., *Escherichia*, *Campylobacter*, and *Helicobacter* (42). It suggests that UPF can benefit the improvement of IBD by reducing *Bacteroidetes* and *Proteobacteria* and increasing *Firmicutes*.

**Figure 8:**
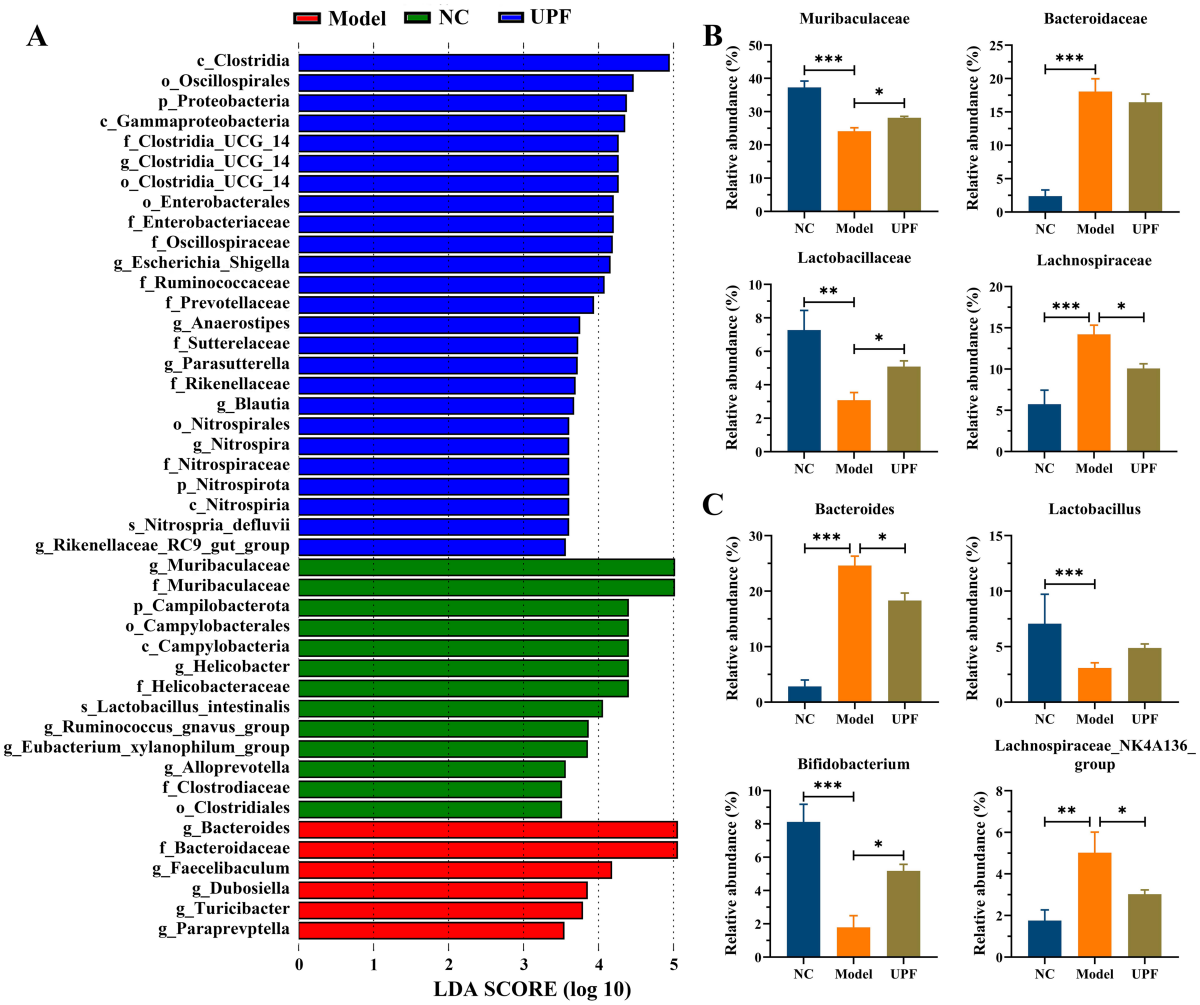
Effect of UPF on gut microbes at the different taxonomic levels (*n* = 5/group). LDA **(A)**. Statistical analysis of key bacteria at the family **(B)** and genus **(C)** level.

LDA showed that there were 44 distinct bacterial taxa across all groups from the phylum level to the genus level (Figure 8A). Among them, 13, 6, and 25 taxa were enriched in the NC, model, and UPF groups. At the family level, *Bacteroidaceae* and *Lachnospiraceae* were distinctly increased in the model group, while *Muribaculaceae* and *Lactobacillaceae* were decreased (Figure 8B). UPF reduced the level of *Lachnospiraceae* and increased the levels of *Muribaculaceae* and *Lactobacillaceae* in mice. At the genus level, *Lactobacillus* and *Bifidobacterium* were distinctly decreased in the model group, and *Lachnospiraceae* NK4A136 group and *Bacteroides* were increased (Figure 8C). UPF increased the levels of *Bifidobacterium* and *Lactobacillus* (*p* > 0.05) and reduced the levels of *Lachnospiraceae* NK4A136 group and *Bacteroides*. *Muribaculaceae*, known as S24-7 family, was reduced in colitis mice, and its level was increased with the improvement of colitis (43). *Lactobacillaceae* species, e.g., *Lactobacillus*, play key roles in host health, which can improve colitis by modulating the cytokine profile and the gut microbiota (44). *Bifidobacterium* is related to appropriate maturation of infant immunity, and some strains can be beneficial for ameliorating colitis symptoms, e.g., *B. bifidum* S17 (45). *Lachnospiraceae* can generate SCFAs that play key roles in human health and disease; however, its correlation with host physiology is always inconsistent across different studies (15, 46). This suggests that effect of UPF on colitis could be associated with an increase in probiotic bacteria and a reduction in harmful bacteria. Notably, the effect of fucoidan or other polysaccharides on the gut microbiota has been widely reported, but the exact mechanism involving alterations of gut microbes lacks systemic research. Fructosan and inulin can create a conducive environment for probiotics in the gut, resulting in a substantial increase in the relative abundance of beneficial bacteria (5, 47). The anaerobic fermentation of polysaccharides in the gut enhances SCFAs production, leading to a reduction in the intestinal pH and the inhibition of pathogenic gram-negative bacteria proliferation in the intestine (48). Moreover, other factors can also influence the level of harmful bacteria, e.g., intestinal barrier function, immunomodulatory effect, antimicrobial peptides, and et al.

### 3.8 UPF modulated abnormality of microbiota metabolites in colitis mice

**Figure 9:**
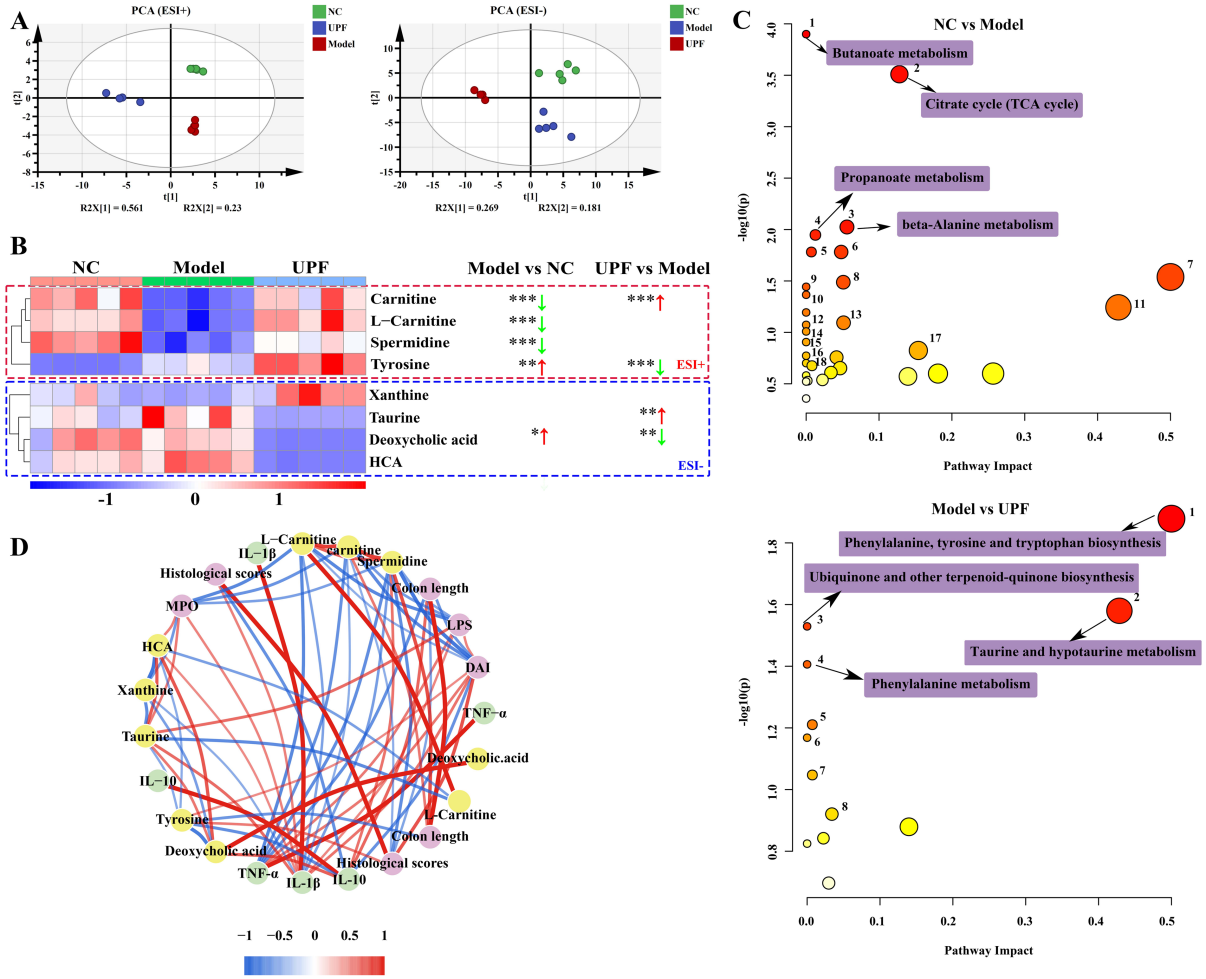
Effect of UPF on microbiota metabolites (*n* = 5/group). PCA based on ESI^+^ and ESI^−^ modes **(A)**. Heatmap constructed by the metabolites that most affected in the different groups **(B)**. Metabolic pathways analysis based on the KEGG database **(C)**. Spearman analysis between key metabolites and biochemical parameters **(D)**.

Although the precise mechanism underlying the effect of gut microbiota on host health remains unclear, one of the primary elements lies in the primary or secondary metabolites produced during microbial metabolism. Therefore, the effect of UPF on the microbiota metabolites was assessed using HPLC/MS (Figure 9). PCA based on ESI^+^ and ESI^−^ modes revealed clear separations between the NC and model groups, implying that the profile of microbiota metabolites was altered with the onset of colitis (Figure 9A). The microbiota metabolites profile was modulated by UPF, and a clear separation was found between the UPF and NC groups. OPLS-DA further exhibited clear differentiation among the NC, UPF, and model groups (Supplementary Figure S2). It indicates that UPF can modulate the alterations in the microbiota metabolites profile but cannot reverse them. This is similar with that tea polysaccharides alleviated colitis in mice by modulating the gut microbiota and metabolites profile (49).

Then, a heatmap was constructed to visualize the metabolites that most affected in the different groups (Figure 9B). Compared to the NC group, the contents of carnitine, L-carnitine, and spermidine were reduced in the model group, and deoxycholic acid and tyrosine were increased. KEGG analysis showed that some pathways were altered with the onset of colitis, particularly butanoate metabolism, alanine metabolism, TCA cycle, and propanoate metabolism (Figure 9C). UPF distinctly increased the contents of carnitine and taurine and decreased the levels of tyrosine and deoxycholic acid. These were associated with the alterations in phenylalanine, tyrosine, and tryptophan biosynthesis, hypotaurine and taurine metabolism, and phenylalanine metabolism. Spearman analysis revealed that spermidine, L-carnitine, and carnitine were negatively correlated with IL-1β, TNF-α, histological score, DAI, MPO, and LPS; deoxycholic acid was positively with IL-1β, TNF-α, MPO, and LPS. Carnitine facilitates the translocation of long-chain fatty acid across mitochondrial membrane for energy generation, beta-oxidation, and elimination of excessive and toxic acyl residues (50, 51). Spermidine is found in all living organisms and involved in maintaining cellular homeostasis, exerting preventive and restorative effect on inflammation (52). Deoxycholic acid exacerbated colitis by triggering activation of NLRP3 inflammasome and production of IL-1β (53). These results suggest that the effect of UPF on colitis can be associated with modulation of the microbiota metabolites, but their exact contribution need more studies.

## 4 Conclusion

This study indicated that FD can enhance host susceptibility to IBD by disrupting the gut barrier integrity and the gut microbiota composition. UPF alleviated colitis that aggravated by FD, which is associated with the preservation of the gut barrier integrity and inhibition of oxidative stress and the MAPK/NF-κB pathway. The underlying mechanism can be associated with modulation of the gut microbiota and metabolites, including an increase in *Bifidobacterium*, spermidine, and carnitine and a reduction in *Bacteroides* and deoxycholic acid. This suggests that UPF holds the potential to serve as a prebiotic dietary fiber to enhance human health. However, some problems need to be further resolved in the future, including the toxicity evaluation of UPF, long-term effect of UPF on colitis or other IBD, the key microbes related to the utilization of UPF and the activities of UPF, and the crucial metabolites that affect the gut health and host health.

## Data Availability

The original contributions presented in the study are included in the article/Supplementary material, further inquiries can be directed to the corresponding author.

## Supplementary material

SUPPLEMENTARY FIGURE S1 Structural information of UPF.

SUPPLEMENTARY FIGURE S2 OPLS-DA models.

## Glossary

AB: Alcian blue
CAT: Catalase
DAI: Disease activity index
DSS: Dextran sulfate sodium
FD: Fiber deficient diet
HE: Hematoxylin and eosin
IBD: Inflammatory bowel disease
iNOS: Inducible nitric oxide synthase
IL-1β: Interleukin-1β
IL-10: Interleukin-10
IκBα: Inhibitors of kappa B kinase α
LDA: Linear discriminant analysis
LPS: Lipopolysaccharide
MDA: Malondialdehyde
MAPK: Mitogen-activated protein kinase
MPO: Myeloperoxidase
NF-κB: Nuclear factor-κb
NO: Nitric oxide
PAS: Periodic acid–Schiff
PCoA: Principal coordinate analysis
ROS: Reactive oxygen species
TJs: Tight junction proteins
TNF-α: Tumor necrosis factor-α
T-SOD: Total superoxide dismutase
UC: Ulcerative colitis
UPF: *Undaria pinnatifida* fucoidan
UPMGA: Unweighted pair-group method with arithmetic means
ZO-1: Zonula occludens-1
