# Supplementary figures and images for: Fucoidan alleviated colitis aggravated by fiber deficiency through protecting the gut barrier, suppressing the MAPK/NF-κB pathway, and modulating gut microbiota and metabolites
Source: Front Nutr. 2025 Jan 24;11:1462584. doi: 10.3389/fnut.2024.1462584 (PMC11802440; doi:10.3389/fnut.2024.1462584)

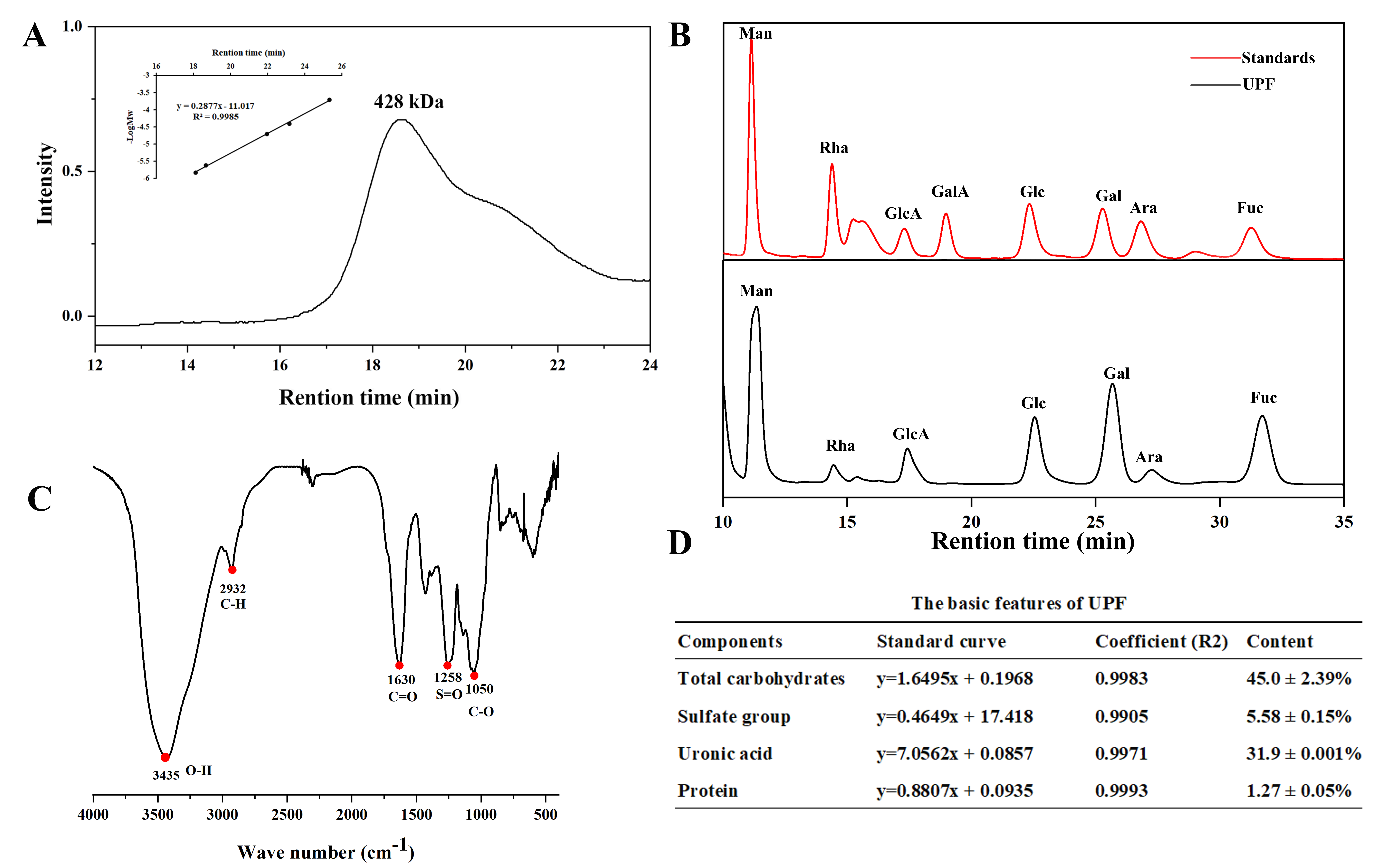

Supplement: SUPPLEMENTARY FIGURE S1 — Structural information of UPF. [file Image_1.PNG]
